# Editorial: Pathogen targeting in chronic respiratory diseases: prevention, therapy and management

**DOI:** 10.3389/fphar.2024.1407616

**Published:** 2024-04-25

**Authors:** Shakti D. Shukla, Kamal Dua, Ronan F. O’Toole

**Affiliations:** ^1^ Discipline of Pharmacy, Graduate School of Health, University of Technology Sydney, Ultimo, NSW, Australia; ^2^ Faculty of Health, Australian Research Centre in Complementary and Integrative Medicine, University of Technology Sydney, Ultimo, NSW, Australia; ^3^ School of Biotechnology, Faculty of Science and Health, Dublin City University, Dublin, Ireland

**Keywords:** respiratory disease, COPD, asthma, COVID-19, IPF

Chronic respiratory diseases (CRDs) are a major cause of morbidity and mortality globally. CRDs, including asthma, chronic obstructive pulmonary disease (COPD), cystic fibrosis and Idiopathic Pulmonary Fibrosis (IPF) are associated with frequent and/or recurrent microbial infections that potentially lead to exacerbations, decreased pulmonary functionality, loss of quality of life, and increased risk of death in patients. Currently available treatments for CRDs focus primarily on symptomatic relief and do not prevent or fully cure the disease. Notably, microbial infections account for a episode of exacerbations of CRDs, however, the specific microbes involved or how they interact with the host are not entirely understood.

This Research Topic examines recent developments in the study of CRDs. The systemic review by Chen et al. determined that CRDs remain one of the foremost disease categories globally. In brief, by utilising data from the Global Burden of Disease Study dataset (GBD 2019), the authors established that there was a 39·8% increase in individuals worldwide with a CRD in 2019 compared to 1990. Furthermore, the investigators reported that “COPD was the leading driver of increased DALY worldwide”. The authors also report that although tobacco smoking is trending downwards, it still remains a major contributor to the prevalence of CRD, and is being joined by risk factor in the form of air pollution, especially in low socio-demographic index regions.

The original article by Xiaofei et al. reports on the ability of erythromycin to inhibit oxidative stress-induced cellular senescence, most likely via the PI3K-mTOR signalling pathway. The authors utilised a combination of cell culture and mouse models for studying the protective effects of erythromycin on cellular senescence, especially via inhibition of P53 and P21 in the CS-induced emphysema mouse model (n = 8 in each experimental group). Moreover, MDA levels significantly increased, and SOD levels decreased in the CS-exposed mice and H2O2-induced BEAS-2B cells. Rapamycin and erythromycin significantly suppressed the expression of P53 and P21. Additionally, rapamycin and erythromycin inhibited the PI3K-mTOR signalling pathway. Whether the PI3K-mTOR pathway can be targeted therapeutically for the management of COPD warrants further investigation in appropriately designed clinical trials.

The review on the role of microbiota in pulmonary health by Marathe et al. provides a timely update on this important topic. The review succinctly summarises the role of the airway microbiome in asthma, and considers how therapeutics, diet, and the environment affect this low biomass community of microbes. Furthermore, the authors discuss how the airway microbiome interacts with the immune system and how it is associated with asthma onset, progression, and severity. Importantly, the authors also examine the implications of pulmonary microbiome in high risk and under-represented groups as well as the role of the gut microbiome in asthma. The up-to-date summary provided by the authors on the pulmonary microbiome in asthma will hopefully encourage more research on the molecular and cellular mechanisms involved in asthma with the potential to lead to new and effective therapeutic interventions.

The original article by Guo et al. reports a novel gene signature (MET, UCHL1, IGF1) that can predict the prognosis in IPF patients with COVID-19. Briefly, the three identified genes are utilised to create a ‘risk signature’ that could be reliably used to predict the prognosis of high-risk and low-risk groups based on the calculated risk score. The findings have crucial significance in providing the treatment/management of IPF patients with COVID-19, which could prove useful in clinical setting when managing IPF patients contracting COVID-19. The study has potential implications for designing similar risk metrices for other respiratory diseases with associated non-pulmonary comorbidities.

It is apparent from this Research Topic that high quality research is being conducted on CRDs. This includes studies to quantify their global burden, to better understand their etiology, and to investigate new ways to prevent and treat these diseases and predict their prognoses.

